# Mutations in *BRCA2* and taxane resistance in prostate cancer

**DOI:** 10.1038/s41598-017-04897-x

**Published:** 2017-07-04

**Authors:** Cathleen Nientiedt, Martina Heller, Volker Endris, Anna-Lena Volckmar, Stefanie Zschäbitz, María A. Tapia-Laliena, Anette Duensing, Dirk Jäger, Peter Schirmacher, Holger Sültmann, Albrecht Stenzinger, Markus Hohenfellner, Carsten Grüllich, Stefan Duensing

**Affiliations:** 1 Molecular Urooncology, Department of Urology, University of Heidelberg School of Medicine, Im Neuenheimer Feld 517, D-69120 Heidelberg, Germany; 2 Department of Medical Oncology, University of Heidelberg School of Medicine, National Center for Tumor Diseases (NCT), Im Neuenheimer Feld 460, D-69120 Heidelberg, Germany; 3 Department of Pathology, University of Heidelberg School of Medicine, Im Neuenheimer Feld 224, D-69120 Heidelberg, Germany; 4 Cancer Therapeutics Program, University of Pittsburgh Cancer Institute, Hillman Cancer Center, 5117 Centre Avenue, Pittsburgh, PA 15213 USA; 5 Cancer Genome Research, National Center for Tumor Diseases, German Cancer Research Center and German Cancer Consortium (DKTK), Im Neuenheimer Feld 460, D-69120 Heidelberg, Germany; 6 Department of Urology, University of Heidelberg School of Medicine, Im Neuenheimer Feld 110, D-69120 Heidelberg, Germany

## Abstract

Mutations in *BRCA1* or *BRCA2* define a subset of prostate cancer patients. Herein, we address the question whether *BRCA1*/*2* mutations have a predictive impact on chemotherapy with docetaxel, a widely used drug in patients with metastatic castration resistant prostate cancer (mCRPC). Fifty-three men treated with docetaxel for mCRPC were tested for somatic *BRCA1*/*2* mutations of the primary tumor. In a subgroup of patients, BRCA1/2 protein expression was tested as a potential surrogate marker for *BRCA1*/*2* inactivation. Eight of 53 patients (15.1%) harbored a deleterious *BRCA2* mutation. No *BRCA1* mutation was found. Patients with a *BRCA2* mutation showed a response rate of 25% to docetaxel in comparison to 71.1% in men with wildtype *BRCA2* (p = 0.019). While the time to develop castration resistance was similar in both subgroups, the overall survival was significantly shorter in patients harboring a *BRCA2* mutation. No correlation between the BRCA1/2 protein expression and the response to docetaxel was found. While the presence of a *BRCA2* mutation does not preclude a response to docetaxel, there is overall a significant correlation between *BRCA2* inactivation and a poor response rate. Our results suggest that a close oncological monitoring of patients with *BRCA2* mutations for taxane resistance is warranted.

## Introduction

Prostate cancer is the most common non-cutaneous cancer and a leading cause of cancer-related mortality in men^1^. There is compelling evidence that genetic factors strongly contribute to the risk of developing prostate cancer. Prominent examples of such risk factors are mutations in the *BRCA1* and *BRCA2* DNA repair genes. Men below the age of 65 carrying a germline mutation in *BRCA1* or *BRCA2* have a 3.4-fold and 8.6-fold, respectively, risk to develop prostate cancer, which make *BRCA2* mutations the strongest known genetic risk factor for prostate cancer^2–4^. A number of studies suggest that prostate cancer patients with germline *BRCA1* or *BRCA2* mutations present at a younger age, have more poorly differentiated tumors and present with a more aggressive clinical course of disease^2, 5–7^. In metastatic castration-resistant prostate cancer (mCRPC), the prevalence of germline mutations in DNA repair genes was 11.8% in a recent study^8^. Several studies have shown a high rate of somatic *BRCA* mutations, in particular in *BRCA2*, in prostate cancer^9–15^. The frequency of somatic *BRCA2* mutations was found to vary between 3% in localized tumors up to 14% in men with mCRPC^9, 10, 14, 15^.

*BRCA1* and *BRCA2* are tumor suppressor genes located on chromosome 17 and chromosome 13, respectively. The *BRCA1* and *BRCA2* genes are structurally unrelated but both function in DNA double strand break (DSB) repair through homologous recombination (HR)^16, 17^. In line with this notion, inactivation of *BRCA1* or *BRCA2* has been found to lead to enhanced mutagenesis and an increase of small indels and copy number alterations (CNAs)^18–20^.

*BRCA1* and *BRCA2* mutations were first reported in women with hereditary breast and ovarian cancer^21^ and have been shown to be associated with sensitivity to poly-(adenosine diphosphate [ADP]-ribose) polymerase (PARP) inhibitors such as olaparib^22^.

A recent phase II trial has shown an overall response rate of 88% in patients with mCRPC harboring deleterious mutations in DNA repair genes and treated with olaparib monotherapy^9^. While these results hold the promise for the first biomarker-driven targeted therapy in prostate cancer, the vast majority of men with mCRPC will continue to receive a taxane-based chemotherapy at some point of time during the course of disease^23^. Notably, there are currently no molecular markers available to predict the response to taxanes.

In this retrospective study, we assessed the frequency and predictive significance of somatic *BRCA1*/*2* mutations for docetaxel monotherapy in 53 men with mCRPC. We found somatic *BRCA2* mutations in the primary tumor in 15.1% of the patients including one patient with a known germline *BRCA2* mutation. The response rate (RR) to docetaxel was 25% in men with a *BRCA2* mutation in comparison to 71.1% in men with wildtype *BRCA2*. While the presence of a *BRCA2* mutations did not preclude a response to docetaxel, our results suggest that close oncological monitoring for taxane resistance is warranted in these patients.

## Patients and Methods

### Patients

In this retrospective study, a total of 53 men were included who were initially diagnosed with locally advanced (≥pT3) or primary metastatic prostate cancer and subsequently were treated with docetaxel for mCRPC at the University of Heidelberg School of Medicine (Table 1). All patients received a prostate biopsy or surgery at the study center between 1998 and 2016. None of these patients had histopathological evidence of neuroendocrine differentiation at the time of diagnosis.

**Table 1:** Patient characteristics (n = 53).

Parameter		
Median age at time of diagnosis, years (range)	63 (40–78)	
Median PSA at diagnosis, ng/mL (range)	30 (0.6–6782)	
Median time to castration resistance, months (range)	22 (2–160)	
Median number of docetaxel cycles (range)	6 (3–12)	
	**n**	**(%)**
**c/pT stage**		
T2	4	(7.5)
T3	37	(69.8)
T4	7	(13.2)
Tx	5	(9.4)
**c/pN stage**		
N0	19	(35.8)
N1	28	(52.8)
Nx	6	(11.3)
**cM stage**		
M0	29	(54.7)
M1	22	(41.5)
Mx	2	(3.8)
Primary metastatic	38	(71.7)
lymph node	16	(30.2)
distant	10	(18.9)
both	12	(22.6)
Localized high-risk (≥pT3)	15	(28.3)
**Risk Group/Gleason Score**		
2 (3 + 4)	5	(9.4)
3 (4 + 3)	5	(9.4)
4 (8)	5	(9.4)
5 (9–10)	37	(69.8)
not available	1	(1.9)
**ECOG status**		
0	30	(56.6)
1	21	(39.6)
2	2	(3.8)
>2	0	(0)
***BRCA1*** **/** ***2*** **mutation status**		
*BRCA1* mutated	0	(0)
*BRCA2* mutated	8	(15.1)
wildtype	45	(84.9)
**Response to docetaxel (≥50% PSA decline)**		
Yes	34	(64.2)
No	19	(35.8)
**Treatment prior to docetaxel**		
Radical prostatectomy	41	(77.4)
Primary radiotherapy	1	(1.9)
Androgen deprivation therapy	53	(100)
Adjuvant radiotherapy	13	(24.5)
Salvage radiotherapy	7	(13.2)
Enzalutamide and/or Abiraterone	8	(15.1)
**Prostate cancer-related death**		
Yes	32	(60.4)
No	14	(26.4)
Alive at last contact	7	(13.2)

All patients and/or their legal guardian(s) provided written informed consent to the study. All experimental protocols and methods were approved under ethics vota 206/2005, 207/2005 and S-085/2012 of the Ethics Committee of the University of Heidelberg School of Medicine. All experiments were carried out in accordance with the June 1964 Declaration of Helsinki (entitled “Ethical Principles for Medical Research Involving Human Subjects”), as last revised, concluded by the World Medical Association.

A response to docetaxel therapy was defined as a reduction in the PSA level of ≥50% at any timepoint during treatment^24^. PSA values immediately before docetaxel treatment and PSA value within two weeks after the last cycle were considered to assess the therapy response.

### Library preparation and semiconductor sequencing

For library preparation, the multiplex PCR-based Ion Torrent AmpliSeq^TM^ technology (Life Technologies) with an FFPE-optimised modified version of the *BRCA1*/*2* community panel (IonTorrent/Thermo Fisher Scientific, Waltham, USA) covering all exons and splice junctions of these genes was used.

Amplicon library preparation was performed with the Ion AmpliSeq Library Kit v2.0 using approximately 10 ng of DNA for each of the three pools. Briefly, the DNA was mixed with each primer pool and the AmpliSeq HiFi Master Mix and transferred to a PCR cycler (BioRad, Munich, Germany)^25^. After the end of the PCR reaction, primer end sequences were partially digested using FuPa reagent, followed by the ligation of barcoded sequencing adapters (Ion Xpress Barcode Adapters, Life Technologies). The final library was purified using AMPure XP magnetic beads (Beckman Coulter, Krefeld, Germany) and quantified using qPCR (Ion Library Quantitation Kit, Thermo Fisher Scientific, Waltham, USA) on a StepOne qPCR machine (Thermo Fisher Scientific, Waltham, USA). The individual libraries were diluted to a final concentration of 100 pM and processed to library amplification on Ion Spheres using Ion PGM™ Template OT2 200 Kit. Unenriched libraries were quality-controlled using Ion Sphere quality control measurement on a QuBit instrument. After library enrichment (Ion OneTouch ES), the library was processed for sequencing using the Ion Torrent PGM HiQ sequencing chemistry and the barcoded libraries were loaded onto a chip, generating a mean coverage of 1000–3000 fold per amplicon.

### Variant Calling and Annotation

Data analysis was performed using the Ion Torrent Suite Software (version 4.4) as described previously^26^. After base calling, the reads were aligned against the human genome (hg19) using the TMAP algorithm within the Torrent Suite. Variant calling was performed with the variant caller plugin within the Torrent Suite Software and the IonReporter package using a corresponding bed-file containing the coordinates of the amplified regions. Only variants with an allele frequency >5% and minimum coverage >200 reads were taken into account. Variant annotation was performed using Annovar^27^. Annotations included information about nucleotide and amino acid changes of RefSeq annotated genes, COSMIC and dbSNP entries as well as detection of possible splice site mutations. For data interpretation and verification, the aligned reads were visualized using the IGV browser (Broad Institute)^28^.

### Immunohistochemistry

Formalin-fixed, paraffin-embedded tissue specimens from a total of sixteen prostate cancers were provided by the tissue bank of the National Center for Tumor Diseases (NCT, Heidelberg, Germany) in accordance with the regulations of the tissue bank and the approval of the ethics committee of the University of Heidelberg School of Medicine. Paraffin sections were deparaffinized in xylene and rehydrated in a graded ethanol series. Antigen retrieval was performed with a steam cooker using retrieval buffer (Target Retrieval Solution, Dako). Primary antibodies were incubated overnight at 4 °C and directed against BRCA1 (clone MS110, Millipore, 1:25), BRCA2 (Sigma, 1:200) and Ki-67 (clone MIB-1, Dako, 1:100). Immunodetection was performed using the Histostain-Plus Detection Kit (3^rd^ Generation, Invitrogen) according to manufacturer’s recommendations. Nuclear counterstaining was provided by hematoxylin (Thermo Scientific).

Tissue specimens were analyzed by two independent observers (C.N. and S.D.). For BRCA1/2 protein expression, five staining categories were defined: negative, weak, partial loss, moderate or strong. Negative, weak and a partial loss of staining were considered as a reduced protein expression and moderate or strong staining as not reduced.

### Statistical analysis

Statistical analyses were conducted with the use of SPSS Statistics 17.0 (SPSS Inc, Chicago, IL). Percentage changes in PSA levels related to docetaxel response was represented in a waterfall plot. Associations of *BRCA2* mutation status or BRCA1/2 protein expression with treatment response to docetaxel and clinico-pathological parameters were statistically analyzed by Fisher’s Exact Test or the Mann-Whitney U test, as appropriate. A p value of ≤0.05 was considered significant.

### Data availability

The datasets generated during the current study are available from the corresponding author on reasonable request.

## Results

### High prevalence of *BRCA2* mutations in patients with high-risk prostate cancer

To investigate the prevalence of *BRCA1*/*2* gene mutations at the time of diagnosis, targeted next generation sequencing (NGS) of tissue specimens obtained through a radical prostatectomy (n = 36), prostate biopsy (n = 13), lymphadenectomy (n = 2) or transurethral resection of the prostate (n = 2) was performed. A deleterious somatic *BRCA2* mutation was found in eight of 53 patients (15.1%; Table 1). One patient carried a known germline *BRCA2* mutation, which was also detected in the primary tumor and has previously been reported^29^. We did not detect any somatic *BRCA1* mutations in this high-risk prostate cancer patient cohort. There was no significant correlation between *BRCA2* mutation status and patient age at diagnosis, ECOG performance status, initial PSA, TNM stage or Gleason score (Suppl. Table 1). The time from diagnosis to castration resistance was 21.9 *versus* 35.5 months for *BRCA2*-mutated and wildtype patients (Suppl. Table 1). This difference of over one year could be due to the aggressive behaviour of *BRCA2*-mutated tumours but also due to the fact that 87.5% of *BRCA2*-mutated cases presented with metastases at diagnosis compared to 68.9% of patients with wildtype *BRCA2* (Suppl. Table 1). We would like to emphasize that we cannot rule out that the lack of statistical significance may be related to the small sample size.

### *BRCA2* mutation status and the patient response to docetaxel

All 53 patients of our cohort developed castration resistance after ADT and subsequently received docetaxel with a median number of treatment cycles of six in both men who were *BRCA2* wildtype (range, 3–12) and in men harboring a *BRCA2* mutation (range, 3–8). The overall RR to docetaxel defined as a PSA decline of ≥50% at any time point during treatment was 64.2% (95% CI, 50.7–75.7%; Table 1).

**Figure 1:**
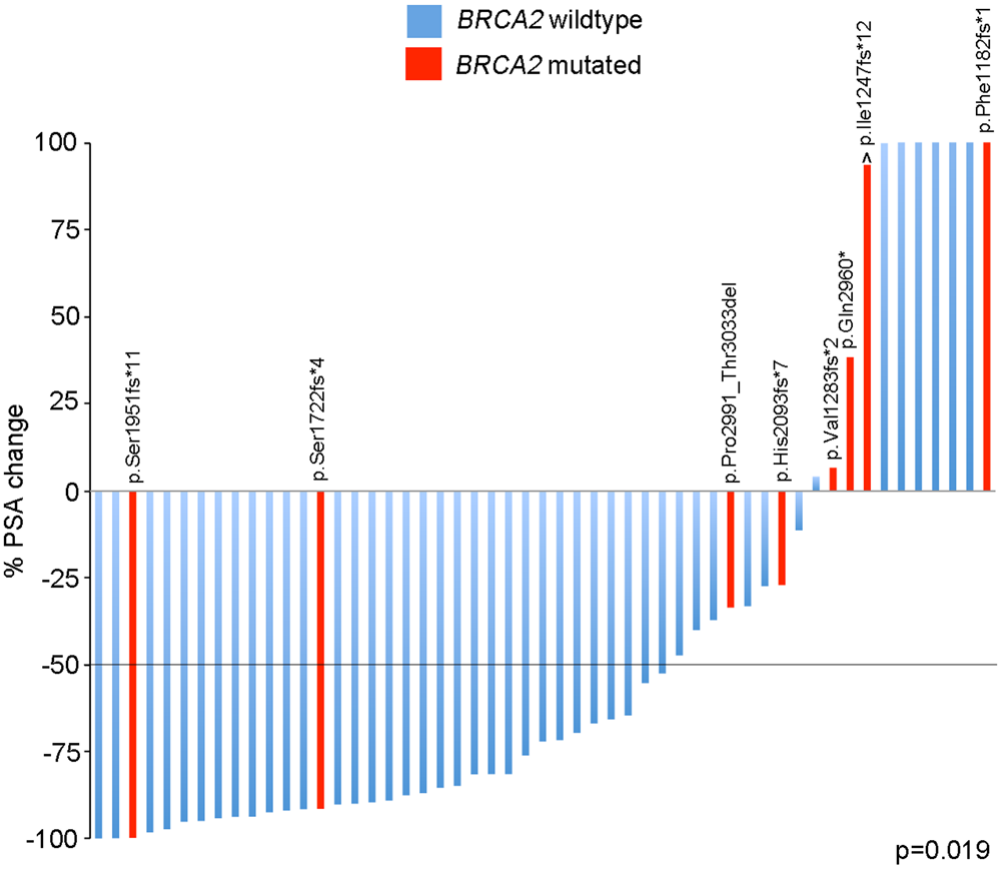
*BRCA2* mutation status and response to docetaxel. Waterfall plot showing the PSA responses (% PSA change) in 53 men with primary metastatic or locally advanced prostate cancer. The dotted line indicates the threshold for defining a PSA response (PSA decline ≥50%). Changes in the protein coding sequence are given for all patients with a *BRCA2* mutation. There was a statistically significant correlation between the presence of a *BRCA2* mutation and the response to docetaxel (p = 0.019, Fisher’s Exact test). The circumflex denotes a patient who carried a known germline *BRCA2* mutations that was also present in the tumor and whose course of disease has previously been reported^29^. The y axis was cut off at 100%.

Of eight patients with a *BRCA2* mutation, two patients showed a response to docetaxel with an >90% PSA decline (RR = 25%; 95% CI, 7.2–59.1%; Fig. 1). However, six of eight patients (75%) showed either a poor response to docetaxel (<50% PSA decline) or a PSA progression (95% CI, 40.9–92.9%; Fig. 1). In comparison, of 45 men who were wildtype for *BRCA2*, 32 showed a PSA response (RR = 71.1%; 95% CI, 56.6–82.3%) whereas 13 patients (28.9%; 95% CI, 17.7–43.4%) showed a poor response or PSA progression under therapy (Fig. 1). The correlation between *BRCA2* mutation status and PSA response to docetaxel was statistically significant (p = 0.019, Fisher’s Exact test; Fig. 1, Suppl. Table 1).

To address the question whether prior treatment may have affected the reponse to docetaxel, we first stratified patients into PSA responders and non-responders and analyzed whether there were any significant differences in the treatment received prior to docetaxel. No significant differences in the prior treatment between PSA responders and non-responders was detected (p > 0.05, Fisher“s Exact test: Suppl. Table 2). In addition, we performed a multivariate logistic regression analysis and found that none of the prior treatment modalities had a significant impact on a favorable PSA response. Variables used were radical prostatectomy (odds ratio [OR] 0.6, p = 0.57), any radiotherapy (OR 1.79, p = 0.42), and enzalutamide and/or abiraterone treatment (OR 0.3, p = 0.16). In this multivariate model, the presence of a *BRCA1*/*2* mutation was negatively associated with a PSA response with borderline significance (OR 0.18, p = 0.065).

Of eight *BRCA2* mutations detected in our patient cohort, six affected exon 11, which encodes the BRC repeat region, whereas two affected the C-terminal DNA binding domain. The two patients with a favorable response to docetaxel both harbored exon 11 mutations, but there was overall no significant correlation between the localization of mutations and the docetaxel response (p > 0.05). One patient with a known *BRCA2* germline mutation showed a PSA progression upon docetaxel treatment (Fig. 1).

**Figure 2:**
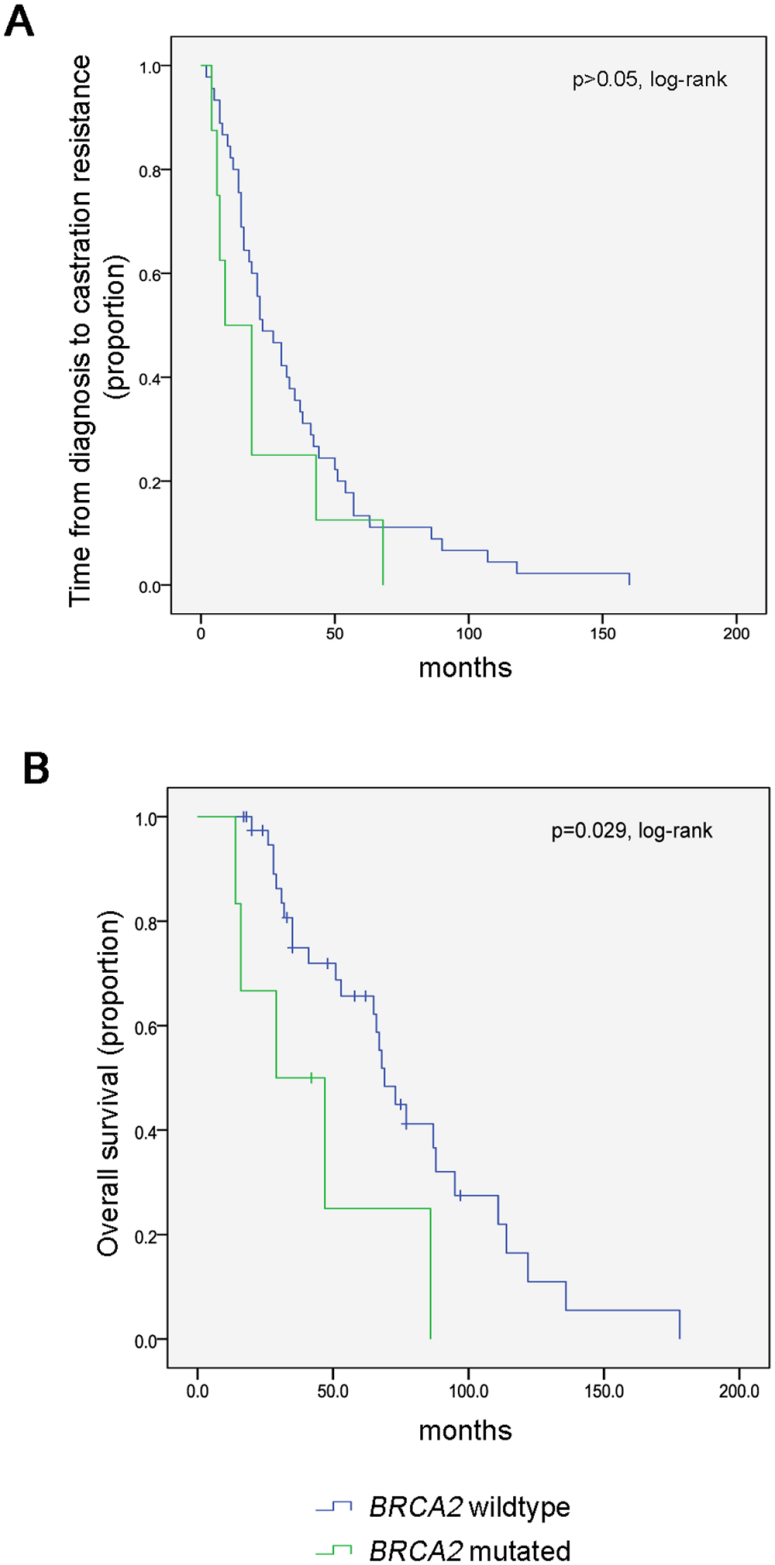
BRCA2 mutation status and patient survival. (**A**,**B**) Kaplan Meier curves showing time to castration resistance in 45 men who were wildtype for *BRCA2* and eight men with a deleterious *BRCA2* mutation (**A**). Overall survival (**B**) was significantly shorter in six men with a *BRCA2* mutation in comparison to 40 men who carried the wildtype gene (p = 0.029; log-rank test). Differences in the patient number in (**B**) are due to the fact that seven men were lost to follow up.

While the time from diagnosis to castration resistance was similar between patients harboring a *BRCA2* mutation in comparison to *BRCA2* wildtype patients (Fig. 2), there was a significantly reduced overall survival in *BRCA2* mutated patients to which the poor response to docetaxel in the majority of patients may have contributed (p = 0.029, log-rank; Fig. 2). However, the small sample size represents a limitation to this conclusion.

Taken together, these results show that *BRCA2* mutations can be detected in a substantial proportion of high-risk prostate cancer patients and that the presence of a *BRCA2* mutation is associated with a poor response to docetaxel in the majority, but not all patients.

### No correlation between *BRCA1*/*2* mutation status and BRCA1/2 protein expression

**Figure 3:**
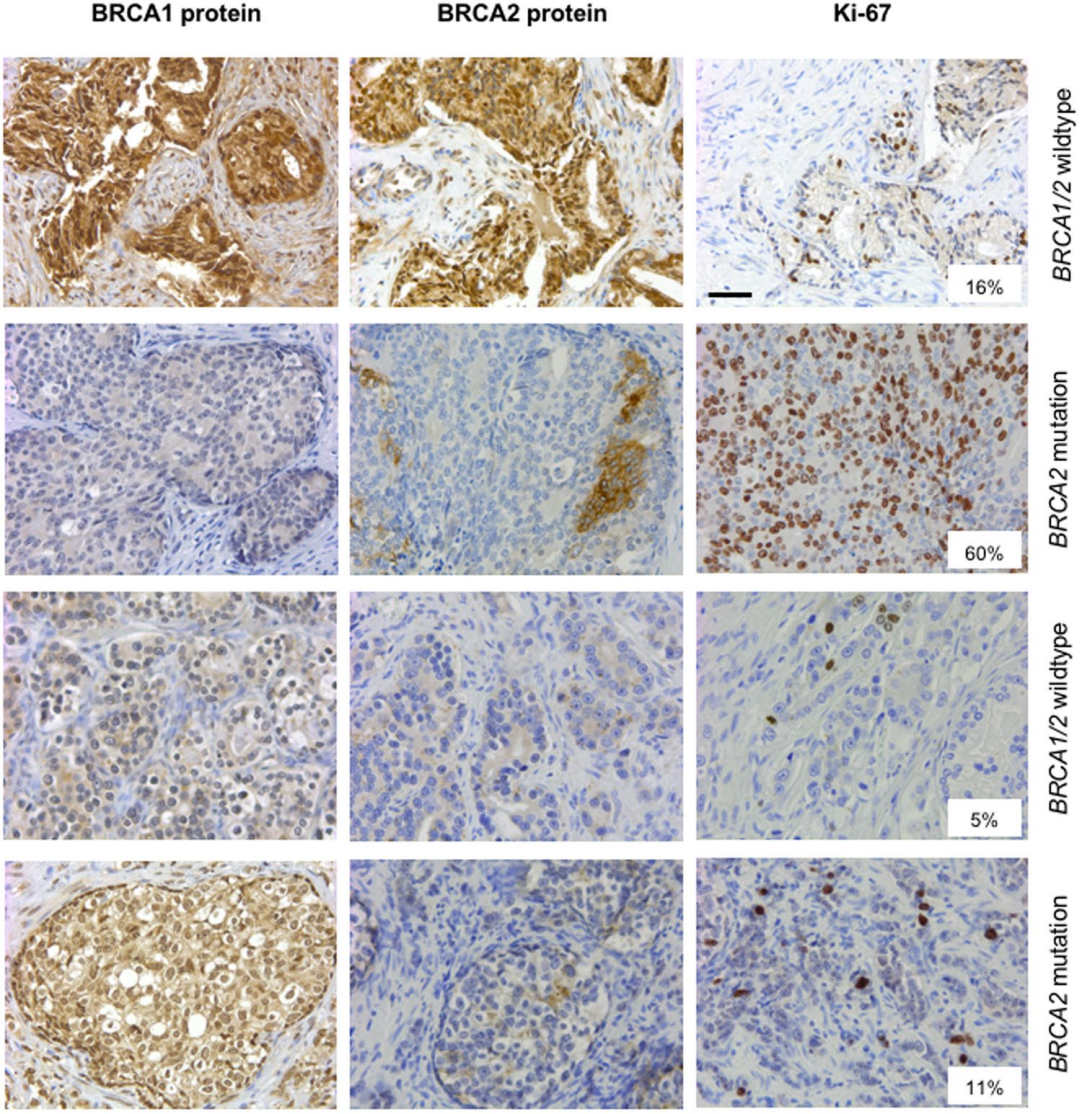
Heterogeneity in BRCA1/2 protein expression and *BRCA1*/*2* mutational status. Immunohistochemical staining for BRCA1, BRCA2 or Ki-67 in four representative tumors. Note that the two *BRCA1*/*2* wildtype tumors showed either a strong nucleocytoplasmic expression of both BRCA1 and BRCA2 or a weak cytoplasmic expression of both proteins. *BRCA2* mutated tumors show a partial loss of BRCA2 protein expression but such a loss was also detectable in *BRCA1*/*2* wildtype tumors (e.g., second row from the bottom). Scale bar = 50 µm.

In order to determine a potential role of BRCA1/2 protein expression as surrogate marker for *BRCA1*/*2* inactivation, tumor specimens of a subgroup of 16 patients selected from our cohort were analyzed by immunohistochemistry (Fig. 3).

BRCA1 and BRCA2 protein expression was seen as predominantly nuclear or nucleocytoplasmic staining in line with previous reports^30, 31^. We found that BRCA2 protein expression was partially lost in some tumors, likely reflecting clonal heterogeneity, a pattern that was not detected for BRCA1.

**Figure 4:**
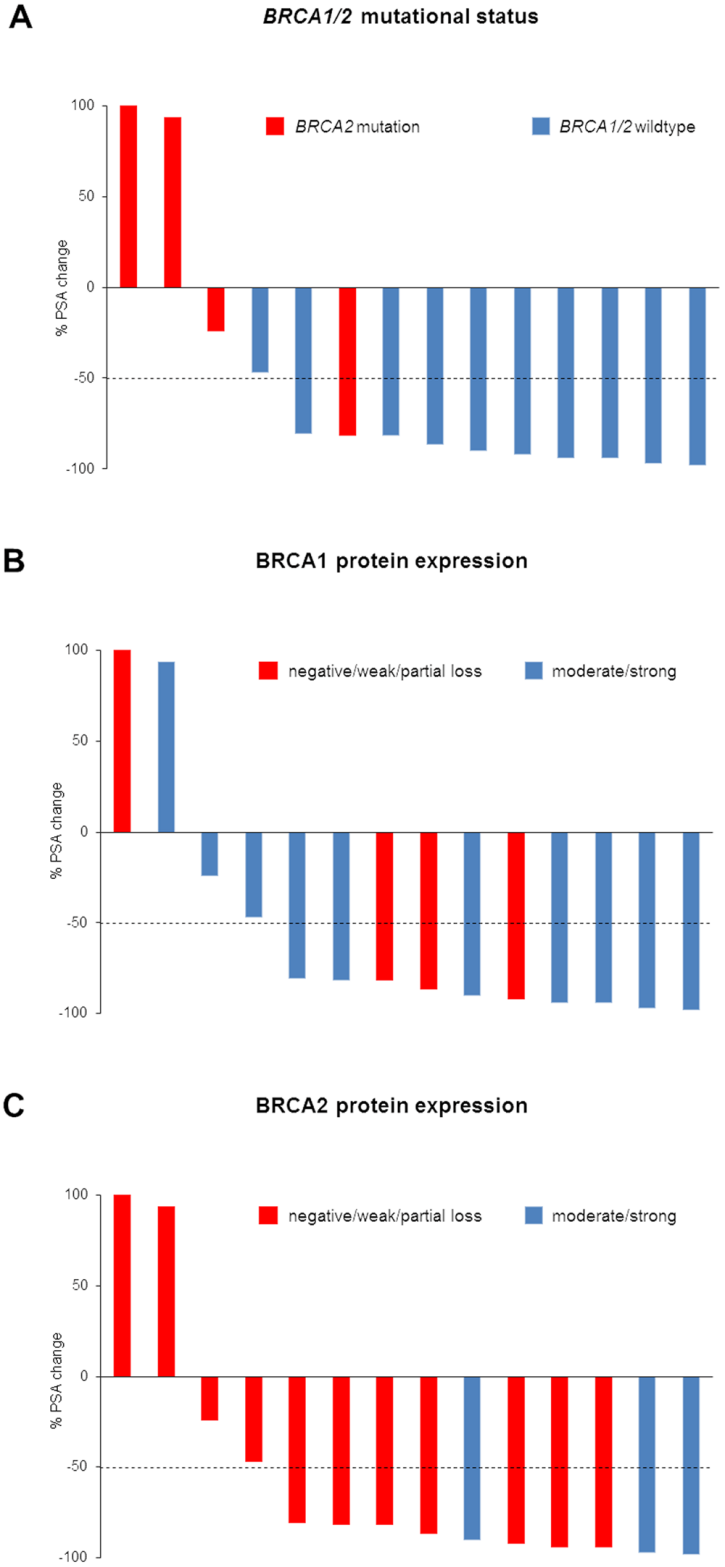
Correlation of *BRCA1*/*2* mutational status or BRCA1/2 protein expression to the PSA response to docetaxel. Waterfall plots for the percentage PSA change after docetaxel treatment stratified into *BRCA1*/*2* mutation status (**A**), BRCA1 protein expression (**B**) or BRCA2 protein expression (**C**). The dotted line indicates the threshold for defining a PSA response (PSA decline ≥50%). The y axis was cut off at 100%.

BRCA1 protein expression was reduced (i.e., negative or weak expression) in five of 16 (31.3%) tumors despite the fact that all tumors were BRCA1 wildtype. A reduction of BRCA2 protein expression (i.e., negative, weak or partial loss of expression) was found in 12 of 16 patients (75%). All five tumor specimens with *BRCA2* mutation had a reduced BRCA2 protein expression, however, a reduced BRCA2 protein expression was also detected in tumors harboring wildtype *BRCA2* (63.6%). There was no statistically significant correlation between *BRCA2* mutation status and BRCA2 protein expression (p > 0.05), nor between *BRCA2* mutation status and BRCA1 protein expression (p > 0.05; Fig. 4).

The median Ki-67 proliferation index across tumors was 12% (range, 3–60%). Two patients with a *BRCA2* mutation showed an excessive proliferation with Ki-67 indices over 50%, however, there was overall no statistically significant correlation between *BRCA2* mutation status and proliferation index. There was also no statistically significant correlation between BRCA1/2 protein expression and clinico-pathological parameters including Gleason score, PSA level at diagnosis, tumor stage, lymph node metastases, distant metastases or the Ki-67 proliferation index (not shown).

In conclusion, BRCA1/2 protein expression is not a suitable surrogate maker for *BRCA1*/*2* inactivation in prostate cancer.

## Discussion

In the present study, we detected *BRCA2* mutations in approximately 15% patients with primary metastatic or localized high-risk prostate cancer who subsequently developed castration resistance and were treated with docetaxel. We show that the presence of a *BRCA2* mutation in the primary tumor negatively affects the RR to docetaxel, which was 25% in *BRCA2*-mutated patients and 71.1% in patients who were wildtype for *BRCA2*. We demonstrate that the heterogeneity of BRCA1/2 protein expression and the lack of concordance with the mutation status precludes the use as a surrogate biomarker for *BRCA1*/*2* inactivation in prostate cancer^32^.

While the small sample size is a limitation of this study, it underscores the role of *BRCA2* not only in the progression prostate cancer but also in the response to one of the current standard therapies.

The key question that arises from our findings is whether a taxane based chemotherapy in prostate cancer patients with a *BRCA2* mutation is the optimal treatment considering our finding that the RR in these patients was only 25%. This proportion is considerably lower than in the *BRCA2* wildtype group presented here or in previous clinical trials where a PSA RR of 50% was reported^33^. Nevertheless, we identified two patients with a deleterious somatic *BRCA2* mutation who showed an ≥90% PSA decline. These findings underscore that patients with a *BRCA2* mutation can have a favorable PSA response and docetaxel resistance may not represent a uniform feature. The exact role of taxane based chemotherapy for the treatment of *BRCA1*/*2* mutated prostate cancer patients has therefore to be further elucidated but our data suggest that patients with known *BRCA1*/*2* mutation should be carefully monitored for PSA response when receiving a taxane based chemotherapy.

A previous study had suggested that *BRCA2* germline carrier status and a response to docetaxel treatment are not mutually exclusive^34^. However, the one patient with a germline *BRCA2* mutation in our study showed a poor response to docetaxel. One caveat of the previous study is that one of the responding patients did receive a combination of docetaxel plus carboplatin and had a significantly longer overall survival than the other patients treated with docetaxel monotherapy. It hence remains unclear, which agent actually lead to the favorable response^34^.

A high response rate to the PARP inhibitor olaparib has been reported in patients with either somatic or germline mutations in *BRCA2*, *ATM* or other genes involved in HR repair^9^. However, some patients may benefit only transiently from such treatment since PARP inhibitor resistance is not uncommon^29, 35^. *BRCA2* inactivation has also been shown to enhance the sensitivity to platinum salts^36^ and, most recently, high-dose testosterone^37^. The increased mutational load associated the *BRCA1*/*2* deficiency^18–20^ may also encourage the use of immune checkpoint blockade in these patients. However, all these alternative treatment modalities, as well as combination therapies such as PARP inhibition in combination with platinum compounds, need to be tested in prospective, multicentric clinical trials, which are so far missing. In addition, a better understanding of the molecular basis of taxane resistance in *BRCA2* mutated prostate cancer is needed for strategies to re-sensitize patients.

BRCA2 has been shown to play a role in a number of mitotic processes including the spindle assembly checkpoint, cytokinesis and daughter cell abcission^38^. A functional spindle assembly checkpoint is critical for taxane-induced cell death. It is hence possible that a defective spindle assembly checkpoint associated with *BRCA2* inactivation causes an impaired efficacy of docetaxel. In addition, a link between *BRCA2* inactivation and multidrug resistance has been reported^39^. Since docetaxel is a substrate for multidrug resistance transporters such as P-glycoprotein, docetaxel efflux may be enhanced in *BRCA2*-mutated prostate cancers. However, since not all *BRCA2*-mutated patients showed an unfavorable response to docetaxel, clonal heterogeneity of the somatic mutation need to be taken into consideration^12^.

There are certain similarities as well as dissimilarities between *BRCA2*-mutated prostate cancer and other tumor entities in which HR defects occur at a sizable proportion such as triple negative breast cancer (TNBC). In TNBCs, docetaxel appears to be less effective than carboplatin in women carrying a *BRCA1*/*2* mutation similar to our findings^40^. TNBCs do not respond to antihormonal treatment. However, in the cohort presented herein, the time to castration resistance was not significantly different between wildtype and patients with a *BRCA2* mutation suggesting that androgen deprivation therapy is not ineffective in the latter subgroup. Whether the RR to next-generation anti-androgens such a abiraterone or enzalutamide differs in *BRCA2*-mutated men in comparison to patients without such a mutation remains to be determined.

Collectively, our results underscore that a substantial proportion of primary metastatic or locally advanced prostate cancer patients who subsequently develop mCRPC harbor a deleterious *BRCA2* mutation. We provide evidence that the majority, but not all, of these patients respond poorly to docetaxel. Clearly, larger studies conducted in a prospective manner are warranted. Given the current lack of these trials, we believe that it would be premature to omit taxanes from the therapeutic armamentarium to treat *BRCA2*-mutated prostate cancer patients. However, close oncological monitoring for docetaxel resistance appears to be necessary.

## Electronic supplementary material

Suppl. Information
