# Supplementary material for: Mutations in BRCA2 and taxane resistance in prostate cancer
Source: Sci Rep. 2017 Jul 4;7:4574. doi: 10.1038/s41598-017-04897-x (PMC5496866; doi:10.1038/s41598-017-04897-x)
Supplement: Supplementary file 1 — Suppl. Information [file 41598_2017_4897_MOESM1_ESM.doc]

**Supplementary Information**

Mutations in *BRCA2* and taxane resistance in prostate cancer

Cathleen Nientiedt, Martina Heller, Volker Endris, Anna-Lena Volckmar, Stefanie Zschäbitz, María A. Tapia-Laliena, Anette Duensing, Dirk Jäger, Peter Schirmacher, Holger Sültmann, Albrecht Stenzinger, Markus Hohenfellner, Carsten Grüllich, and Stefan Duensing

**Suppl. Table 1. Correlation between *BRCA2* mutational status and clinicopathological patient characteristics (n=53).**

|  | ***BRCA2* wildtype** | |  | ***BRCA2* mutated** | | **p value** |
| --- | --- | --- | --- | --- | --- | --- |
|  |  |
|  | (n=45; 84.9%) | |  | (n=8; 15.1%) | |  |
|  |  | |  |  | |  |
| **Age at diagnosis, years, mean (SD)** | 61.7 | (8.1) |  | 66.6 | (7.2) | n.s. |
| **PSA at diagnosis, ng/ml, mean (SD)** | 262.3 | (1019) |  | 69.2 | (121.3) | n.s. |
| **Time to castration resistance, month, mean (SD)** | 35.5 | (32.8) |  | 21.9 | (22.5) | n.s. |
| **Number of docetaxel cycles, mean (SD)** | 5.9 | (1.9) |  | 5.6 | (1.5) | n.s. |
|  |  |  |  |  |  |  |
|  | **n** | **(%)** |  | **n** | **(%)** |  |
| **c/pT stage (n, %)** |  |  |  |  |  |  |
| T2 | 4 | (8.9) |  | 0 | (0) | n.s. |
| T3 | 30 | (66.7) |  | 7 | (87.5) |  |
| T4 | 6 | (13.3) |  | 1 | (12.5) |  |
| Tx | 5 | (11.1) |  | 0 | (0) |  |
|  |  |  |  |  |  |  |
| **c/pN stage (n, %)** |  |  |  |  |  |  |
| N0 | 17 | (37.8) |  | 2 | (25) | n.s. |
| N1 | 22 | (48.9) |  | 6 | (75) |  |
| pNx | 6 | (13.3) |  | 0 | (0) |  |
|  |  |  |  |  |  |  |
| **cM stage (n, %)** |  |  |  |  |  |  |
| M0 | 25 | (55.6) |  | 4 | (50) | n.s. |
| M1 | 18 | (40) |  | 4 | (50) |  |
| Mx | 2 | (4.4) |  | 0 | (0) |  |
|  |  |  |  |  |  |  |
| **Primary metastatic cancer** |  |  |  |  |  |  |
| lymph node | 13 | (28.9) |  | 3 | (37.5) | n.s. |
| distant | 7 | (15.6) |  | 1 | (12.5) |  |
| both | 11 | (24.4) |  | 3 | (37.5) |  |
| all | 31 | (68.9) |  | 7 | (87.5) |  |
|  |  |  |  |  |  |  |
| **Localized high-risk** | 14 | (31.1) |  | 1 | (12.5) |  |
|  |  |  |  |  |  |  |
| **Risk group/Gleason score (n, %)** |  |  |  |  |  |  |
| 2 (3+4) | 3 | (66.7) |  | 2 | (25) | n.s. |
| 3 (4+3) | 4 | (8.9) |  | 1 | (12.5) |  |
| 4 (8) | 5 | (11.1) |  | 0 | (0) |  |
| 5 (9-10) | 32 | (71.1) |  | 5 | (62.5) |  |
| not available | 1 | (2.2) |  | 0 | (0) |  |
|  |  |  |  |  |  |  |
| **ECOG status** |  |  |  |  |  |  |
| 0 | 27 | (60) |  | 3 | (37.5) | n.s. |
| 1 | 16 | (35.6) |  | 5 | (62.5) |  |
| 2 | 2 | (4.4) |  | 0 | (0) |  |
| >2 | 0 | (0) |  | 0 | (0) |  |
|  |  |  |  |  |  |  |
| **Response to docetaxel (≥50% PSA decline)** |  |  |  |  |  | p=0.019 |
| Yes | 32 | (71.1) |  | 2 | (25) |  |
| No | 13 | (28.9) |  | 6 | (75) |  |
|  |  |  |  |  |  |  |
| **Prostate cancer-related death**  Yes |  |  |  |  |  |  |
| Yes | 27 | (60) |  | 5 | (62.5) | n.s. |
| No | 12 | (26.7) |  | 2 | (25) |  |
| Alive at last contact | 6 | (13.3) |  | 1 | (12.5) |  |
|  |  |  |  |  |  |  |

**Suppl. Table 2. Correlation between the response to docetaxel**

**and prior treatment (n=53).**

|  | ***≥50% PSA response*** | |  | ***<50% PSA response*** | | **p value** |
| --- | --- | --- | --- | --- | --- | --- |
|  |  |
|  | (n=34; 64.2%) | |  | (n=19; 35.8%) | |  |
|  | n | (%) |  | n | (%) |  |
|  |  |  |  |  |  |  |
| **Radical prostatectomy** |  |  |  |  |  | n.s. |
| yes | 25 | (73.5) |  | 16 | (84.2) |  |
| no | 9 | (26.5) |  | 3 | (15.8) |  |
|  |  |  |  |  |  |  |
| **Primary radiotherapy** |  |  |  |  |  | n.s. |
| yes | 0 | (0) |  | 1 | (5.3) |  |
| no | 34 | (100) |  | 18 | (94.7) |  |
|  |  |  |  |  |  |  |
|  |  |  |  |  |  |  |
| **Adjuvant radiotherapy** |  |  |  |  |  | n.s. |
| yes | 10 | (29.4) |  | 3 | (15.8) |  |
| no | 24 | (70.6) |  | 16 | (84.2) |  |
|  |  |  |  |  |  |  |
| **Salvage radiotherapy** |  |  |  |  |  | n.s. |
| yes | 5 | (14.7) |  | 2 | (10.5) |  |
| no | 29 | (85.3) |  | 17 | (89.5) |  |
|  |  |  |  |  |  |  |
| **Enzalutamide/Abiraterone** |  |  |  |  |  | n.s. |
| yes | 3 | (8.8) |  | 5 | (26.3) |  |
| no | 31 | (91.2) |  | 14 | (73.7) |  |
|  |  |  |  |  |  |  |
| ***BRCA1/2* mutated** |  |  |  |  |  | p=0.019 |
| yes | 2 | (5.9) |  | 6 | (31.6) |  |
| no | 32 | (94.1) |  | 13 | (68.4) |  |
|  |  |  |  |  |  |  |
|  |  |  |  |  |  |  |
|  |  |  |  |  |  |  |

*All patients received androgen deprivation therapy.
